# Biceps Tenodesis for the Treatment of Type II Superior Labral Anterior Posterior (SLAP) Tears in Overhead Athletes Under the Age of 35: A Case Series

**DOI:** 10.7759/cureus.71141

**Published:** 2024-10-09

**Authors:** Hans Drawbert, Kelsey Schnackenberg, Michael Obermeier, Marc Tompkins

**Affiliations:** 1 Sports Medicine, Florida International University Herbert Wertheim College of Medicine, Miami, USA; 2 Orthopedics, TRIA Orthopedics, Bloomington, USA; 3 Orthopedic Surgery, University of Minnesota Medical School, Minneapolis, USA

**Keywords:** overhead athletes, shoulder surgery, slap injury, slap repair, tenodesis

## Abstract

Background: The best treatment for type II superior labral anterior posterior (SLAP) tears in overhead athletes is not well defined.

Questions/purpose: The purpose of this study was to examine post-surgical outcomes in overhead athletes under the age of 35 who underwent primary biceps tenodesis for an isolated type II SLAP tear. We hypothesized that these patients would have high rates of return to play, as well as recovery of range of motion (ROM) and strength after surgery.

Patients and methods: Patients were between the ages of 18 and 35, had a primary isolated type II SLAP tear confirmed on magnetic resonance imaging (MRI), and were injured performing overhead activities. All patients underwent biceps tenodesis using an arthroscopic suprapectoral approach. Patients underwent standard postoperative rehabilitation lasting up to one year. Function and outcomes were measured at baseline, three months, six months, one year, and two years using range of motion, strength testing, and patient-reported outcomes (PROs) (Western Ontario Shoulder Instability Index (WOSI), Kerlan-Jobe Orthopedic Clinic Score (KJOC), American Shoulder and Elbow Surgeons Assessment Form (ASES), and Single Assessment Numeric Evaluation (SANE)).

Results: Five patients were included in the case series. There was consistent improvement at each time point on PROs: WOSI, p=0.01; KJOC, p=0.04; SANE, p=0.02; and ASES, p=0.03. Range of motion increased from baseline to each time point with a significant improvement in forward flexion (p=0.03). In strength testing, there were improvements in all exercises and a significant improvement in abducted external rotation between years 1 and 2 (p<0.01).

Conclusions: This study demonstrated that biceps tenodesis in overhead athletes under the age of 35 provides improved outcomes, ROM, and strength.

## Introduction

Superior labral anterior posterior (SLAP) tears are a common pathology seen in patients with shoulder injuries. In the general public, SLAP tears can be seen in estimates of up to 25% during arthroscopic shoulder surgery and are reported at different rates for different populations [1]. Professional baseball players have some of the highest rates of SLAP tears; Chalmers et al. [2] found that from 2012 to 2016, there were 581 shoulder surgeries done in professional baseball players, and 27% of these involved the superior labrum. In a review of National Football League (NFL) players, it was found that SLAP tears comprised a smaller amount of 3.1% of shoulder injuries over the years 2000-2014, with the highest incidence being in offensive linemen (28%) [3]. SLAP tears are seen in military personnel at higher rates than in the general public as well [4]. While different populations show different rates of SLAP tears, it has been well established that SLAP tears are common and especially affect individuals involved in overhead activities.

SLAP tears are treated in a variety of ways based on patients' age, level of activity, type of SLAP tear, surgeon's preference, and success of conservative treatment, among other variables [1,5,6]. The traditional paradigm to surgically treat SLAP tears has been to perform a SLAP repair in younger, more active athletes and biceps tenodesis or tenotomy in patients older than 35 [5,7]. However, SLAP repairs have become a topic of debate, especially for individuals involved in overhead activities. In a systematic review by Gorantla et al. [8], the percentage of patients who reported a good or excellent outcome following SLAP repair ranged from 40% to 94%, with a reported return to previous level of play ranging from 20% to 94%. Some reasons associated with low rates of satisfaction in patients who underwent SLAP repair are chronic pain, stiffness, need for revision, and inability to return to previous levels of activity [8-10]. With this variability in outcomes, there has been work done to see if biceps tenodesis would be a viable alternative surgical option for SLAP tears. Recent literature has shown that a high percentage of patients have successful outcomes following biceps tenodesis for SLAP tear, and some literature even demonstrates superior outcomes in these patients for biceps tenodesis compared to SLAP repair [6,7,10-14]. A study by Boileau et al. [15] found that 87% of their patients who had a biceps tenodesis returned to prior level of activity compared to only 20% of patients who had a SLAP repair. A systematic review by de SA et al. [11] found that return to play in patients who underwent biceps tenodesis was 73%-100% when compared with 20%-95% of patients undergoing SLAP repair [11].

There are a small number of studies that have looked specifically at biceps tenodesis treatment for young patients with SLAP tears. In an active military population with a mean age of 42.6, Provencher et al. [16] found that biceps tenodesis resulted in improvement in outcome scores with a high level of return to activity for patients with biceps pathology. Griffin et al. [12] evaluated patients under the age of 25 who underwent biceps tenodesis for biceps pathology that was not isolated to SLAP tears, and they also found an improvement in outcomes and return to activity. More data is needed for young patients with isolated type II SLAP tears who undergo primary biceps tenodesis.

The purpose of this study is to examine post-surgical outcomes in overhead athletes under the age of 35 who underwent primary biceps tenodesis for an isolated type II SLAP tear. We hypothesize that their return to play, patient-reported outcome (PRO) scores, and levels of stiffness and strength will be satisfactory when compared with similar patients in the literature who underwent SLAP repair.

## Materials and methods

The study "SLAP Repair Versus Biceps Tenodesis for SLAP Tears in the Shoulder" (IRB code #: 1401M47421) was approved through the University of Minnesota's Institutional Review Board, and all patients gave their consent to participate in the study.

Patient selection

Patients were prospectively enrolled based on the following inclusion criteria: age between 18 and 35 at the time of consent and patients who had a primary isolated type II SLAP tear that was confirmed on magnetic resonance imaging (MRI). Patients were excluded if they had any of the following: a concomitant procedure that was needed (anterior or posterior labral repair, rotator cuff repair, and distal clavicle excision), revision surgery, previous shoulder surgery, major medical illness, or inability to speak or read English.

Overhead athletes include anyone who is involved in hitting or throwing overhead in their sports. Examples include athletes in baseball, softball, volleyball, tennis, lacrosse, etc.; this would also include specialty positions such as quarterback or anything that requires overhead motion and extremity control in that sports activity. Demographic and clinical information from the medical records of subjects who participated in this study were collected. This included age, sex, laterality, date of surgery, primary and secondary sports played, and method of injury.

Surgical procedures

All patients underwent a biceps tenodesis procedure, performed in the same way by two surgeons. This was done using an arthroscopic suprapectoral approach. A tunnel was drilled on the anterior aspect of the humerus in line with the normal trajectory of the biceps tendon. This was followed by fixation of the biceps tendon into the tunnel using a biocomposite interference screw [17]. All tenodesis sites were distal to the bicipital groove and roughly at the level of the superior border of the pectoralis major tendon. All surgeries were performed at a single outpatient orthopedic surgical center.

Rehabilitation

All patients underwent a standard postoperative rehabilitation protocol. This involved three phases: 0-4 weeks, 4-12 weeks, and 3-12 months. The first phase consisted of using a sling for comfort and performing elbow and shoulder range of motion (ROM) exercises without resistance. In the second phase, patients no longer used a sling and started doing light isometric exercises. After three months, patients could start sport-related rehabilitation and strength exercises.

Assessment tools

Patients were evaluated using outcome instruments, as well as evaluated for active range of motion (AROM) and isometric strength testing at baseline, six months, one year, and two years. Surgeons and subjects were unblinded, but the ROM and strength testing examinations were performed by a blinded, trained examiner. Strength and ROM examinations were conducted by a blinded examiner, and subjects were instructed to refrain from discussing their procedure with the examiner. The examiner was blinded from any documentation of the surgical procedure in the electronic medical record, and the participant wore a shirt or gown covering both shoulders during testing for blinding of the surgical shoulder/surgical incision.

Patients completed the Western Ontario Shoulder Instability Index (WOSI), Kerlan-Jobe Orthopedic Clinic Score (KJOC), American Shoulder and Elbow Surgeons Assessment Form (ASES), and Single Assessment Numeric Evaluation (SANE). The baseline measures were completed by participants following informed consent and prior to surgery. AROM measurements included flexion, abduction, external rotation at 0 and 90 degrees abduction, and internal rotation at 90 degrees abduction. The measurements were taken using a goniometer (Baseline® HiRes™ Plastic Absolute+Axis™ Goniometer, 360 degree head, 12 inch arms). Isometric strength testing was done using a handheld dynamometer in shoulder forward flexion, shoulder external rotation at 90 degrees abduction, shoulder internal rotation at 90 degrees abduction, elbow flexion, and supination. The measurements were taken using a handheld dynamometer (IsoForce Control Dynamometer; MDS Medical Device Solutions AG, Bern, Switzerland).

Data collection

Data was collected at the time of visit for each patient. Surveys were filled out and collected at the assigned pre- and postoperative appointments. Range of motion and strength testing occurred at these visits as well. When patients were unable to complete a visit and the accompanying tests, we continued to collect data at the following visits.

Statistical analysis

Data was analyzed using Microsoft Excel (Microsoft Corp., Redmond, WA). Data distribution was tested using a Shapiro-Wilk test. Comparison between baseline data and any follow-up time points was completed using a two-tailed T-Test; the significance level for the p-value was set at <0.05.

## Results

Demographics

All patients were under the age of 35 and overhead athletes. Of five patients, four had injured their right shoulder and one had injured their left; these all corresponded to the dominant arm of the athletes. The overhead sports played were baseball, softball, ultimate frisbee, soccer (goalie), and rock climbing. Based on their clinical reporting, all patients had returned to the same level of sports postoperatively. All data was found to be normally distributed. Patient-specific information is displayed in Table 1.

**Table 1:** Demographics N/A: not available/applicable

Age	Sex	Hand dominance	Injured side	Sport	Position	Athletic level
28	Female	Right	Right	Softball	Catcher	Recreational weekly or greater
31	Female	Left	Left	Ultimate frisbee	Handler	National club level
28	Female	Right	Right	Soccer	Goalie	Recreational weekly or greater
20	Male	Right	Right	Baseball	Pitcher	Division I college
24	Male	Right	Right	Rock climber	N/A	Recreational weekly or greater

Patient-reported outcomes

For all patient-reported outcomes, there was consistent improvement at each time point (Table 2).

**Table 2:** Patient-reported outcomes Average patient-reported outcome scores of participants and percent change from baseline. WOSI: Western Ontario Shoulder Instability Index, KJOC: Kerlan-Jobe Orthopedic Clinic Score, SANE: Single Assessment Numeric Evaluation, ASES: American Shoulder and Elbow Surgeons Assessment Form

	Baseline	6 months			1 year			2 years		
	Average	Average	% change	P-value	Average	% change	P-value	Average	% change	P-value
WOSI	1193.00	312.50	73.80%	0.01	256.67	78.49%	0.05	109.00	90.86%	<0.01
KJOC	324.80	659.25	103.00%	0.04	733.33	125.78%	0.04	778.50	139.69%	0.24
SANE	48.00	82.50	71.87%	0.02	89.00	85.42%	0.15	92.50	92.71%	0.08
ASES	57.10	91.38	60.02%	0.03	95.89	67.93%	0.15	98.50	72.50%	0.13

All four outcome scores saw significant improvement from baseline to six months: WOSI, from 1193 to 313 (p=0.01); KJOC, from 325 to 659 (p=0.04); SANE, from 48 to 83 (p=0.02); and ASES, from 57 to 91 (p=0.03). There was a significant improvement in KJOC from 659 at six months to 733 at one year (p=0.04). There was a significant improvement in WOSI from 256.67 in one year to 109.00 in two years (p<0.01). Based on the WOSI sports/recreation and lifestyle sections, all but one patient had returned to a similar activity level by one year.

Active range of motion testing

Active range of motion increased in all five movements from baseline to each time point (Table 3). 

**Table 3:** Active range of motion testing Average active range of motion in degrees for given exercises and percent change from baseline. ER: external rotation, IR: internal rotation

	Baseline	6 months			1 year			2 years		
	Average	Average	% change	P-value	Average	% change	P-value	Average	% change	P-value
Forward flexion	157.60	168.00	6.60%	0.03	172.67	9.56%	0.31	175.00	11.04%	0.50
Abduction	155.40	176.25	13.42%	0.05	178.33	14.76%	0.40	180.00	15.83%	0.47
ER at 0	69.80	75.00	7.45%	0.55	76.67	9.84%	0.59	84.00	20.34%	0.42
ER at 90	100.00	96.25	-3.75%	0.39	96.67	-3.33%	1.00	95.00	-5.00%	0.50
IR at 90	43.60	62.50	43.35%	0.18	58.33	33.79%	0.39	62.50	43.35%	0.19

There was a significant improvement in forward flexion from 158 degrees at baseline to 168 degrees at six months (p=0.03).

Isometric strength testing

In the isometric strength testing exercises, there were improvements in all exercises from baseline to each time point; the only exception was abducted external rotation from baseline to six-month follow-up (Table 4).

**Table 4:** Isometric strength testing Average strength testing in Nm for given exercises and percent change from baseline. Isometric strength is measured using a handheld dynamometer. ER: external rotation, IR: internal rotation

	Baseline	6 months			1 year			2 years		
	Average	Average	% change	P-value	Average	% change	P-value	Average	% change	P-value
ER at 90	51.31	47.78	-6.89%	0.98	73.83	43.90%	0.08	75.63	47.39%	<0.01
IR at 90	55.44	66.18	19.36%	0.22	85.27	53.80%	0.35	101.38	82.86%	0.36
Supination	47.94	60.03	25.21%	0.34	56.50	17.86%	0.38	61.85	29.02%	0.21
Forward flexion thumb down	43.73	53.23	21.71%	0.53	57.65	31.83%	0.43	65.63	50.07%	0.53
Forward flexion thumb up	47.10	51.15	8.60%	0.60	57.18	21.41%	0.45	71.95	52.76%	0.29
Elbow flexion	118.12	122.89	4.04%	0.14	135.93	15.08%	0.98	166.48	40.94%	0.34

There was a significant improvement in abducted external rotation from 73.83 at one year to 75.63 at two years (p<0.01).

## Discussion

SLAP tears are a common shoulder pathology, especially in overhead athletes. They are challenging injuries to treat, and there is no clear consensus on which is a better surgical approach, SLAP repair or biceps tenodesis. All patients in our study involving young overhead athletes undergoing biceps tenodesis for a type II SLAP tear had continuous improvement from preoperative status to the two-year follow-up for patient-reported outcomes, range of motion, and strength testing. There was no regression for the patients in any of the outcome metrics, and the return to activity level was equal or similar to that before injury. These results suggest that performing biceps tenodesis in younger patients, and specifically overhead athletes, provides a viable option for treating type II SLAP tears.

There are several recent studies that have looked at similar patient populations; while they have not involved all of the parameters in our study in terms of prospective data for isolated type II SLAP tears in young overhead athletes, the results have demonstrated good outcomes. Griffin et al. [12] showed that patients under the age of 25 who underwent biceps tenodesis had improved ASES scores (54.7 to 81.7), ASES functional scores (17.5 to 25.1), and simple shoulder test scores (7.4 to 10.1) (p<0.01). In a study of active military personnel who underwent biceps tenodesis for SLAP tears or biceps tendonitis, there was improved Western Ontario Rotator Cuff Index (WORC) and SANE scores, as well as return to full activity of 82%; however, their average age was higher at 42.6 years (range: 26.3-56.5 years) [13]. A systematic review conducted by Frantz et al. [18], with eight included studies, evaluated outcomes in overhead athletes with SLAP tears who underwent biceps tenodesis. They found that ASES scores were between 82 and 97, 12-item short-form health survey (physical) went from 50 to 54, VAS pain scores were from 0.8 to 1.5, KJOC scores were from 66 to 79, satisfaction ranged from 80% to 7%, and the overall return to sports was 70% (60 out of 86). Our results are consistent with these studies, that performing biceps tenodesis on younger patients can offer satisfactory and improved postoperative outcomes and return to play. Our study adds to the previous literature because it prospectively followed and evaluated only overhead athletes under the age of 35 with type II SLAP tears and found similar or better outcomes to other studies; this information adds insight to treating the challenging patient population of young, overhead athletes with type II SLAP tears.

Range of motion is an important aspect of function following shoulder surgery and has not been well reported in previous studies of biceps tenodesis for overhead athletes. Our study evaluated five aspects of range of motion: forward flexion, abduction, external rotation, external rotation at 90 degrees of abduction, and internal rotation at 90 degrees of abduction. At the two-year follow-up, the patients were found to have either an increased range of motion or a consistent range of motion from their baseline. There was no regression in the range of motion in any of the measurements throughout the study period, suggesting that there was no increased stiffness following surgery. These range of motion results are similar to or better than the range of motion results in other studies for patients who underwent SLAP repair or biceps tenodesis. A systematic review by de SA et al. [11] compared outcomes in SLAP repair and biceps tenodesis patients. They found that at the final follow-up, forward flexion was 175 and external rotation was 65 for tenodesis patients and between 159 and 176 for forward flexion and external rotation of 51-97 in repair patients. Their values are very similar to ours, with our patients having a forward flexion of 175 and an external rotation of 84 at the final follow-up. In a study of only biceps tenodesis patients, forward elevation at two-year follow-up was found to be 169 degrees [19]. In these studies, the tenodesis patients were older, but the SLAP repair patients were more similar in age. The fact that the results in our young patients were similar to or better than similar age SLAP repair patients and older biceps tenodesis patients demonstrates that good range of motion outcomes can be expected in young overhead athletes undergoing biceps tenodesis.

Strength is another important aspect of function following shoulder surgery that lacks reporting in previous studies of biceps tenodesis for overhead athletes. We evaluated six strength measurements: external rotation, internal rotation, supination, forward flexion thumb down, forward flexion thumb up, and elbow flexion. There was an improvement from baseline to the final follow-up of 47% in external rotation, 83% in internal rotation, 29% in supination, 50% in forward flexion thumb down, 53% in forward flexion thumb up, and 41% in elbow flexion. Restoration or improvement in strength following biceps tenodesis is similar to other biceps tenodesis studies not dedicated to overhead athletes. Boileau et al. [15] showed no difference in the strength of elbow flexion and supination between SLAP repair patients and tenodesis patients at the final follow-up (mean of 35 months). In another study, Friedman et al. [20] compared tenodesis to tenotomy and found no difference in elbow flexion between groups. In all exercises in our study, patients demonstrated improvements at each successive time point, and at the final follow-up, they had higher strength scores than at baseline, which demonstrates that good strength outcomes can be expected in young overhead athletes undergoing biceps tenodesis.

Limitations

This study is not without limitations. The first and foremost limitation of this study was the small sample size. We only had five patients who underwent biceps tenodesis in the study, which limits the statistical power. It is possible that more of the findings would have been statistically significant with a greater sample size. Also, this study was performed at only one institution; therefore, it may not be reflective of all overhead athlete patients with type II SLAP tears. Both positive and negative in terms of limitations, the patients were involved in different overhead sports.

## Conclusions

This study demonstrated improved patient-reported outcomes, range of motion, and strength testing following biceps tenodesis surgery with the majority of patients returning to their pre-injury activity levels, which ranged from weekly recreational to division I college. The data suggest that biceps tenodesis is a viable option for overhead athletes under the age of 35 with type II SLAP tears.

## References

[1] Knesek M, Skendzel JG, Dines JS, Altchek DW, Allen AA, Bedi A (2013). Diagnosis and management of superior labral anterior posterior tears in throwing athletes. Am J Sports Med.

[2] Chalmers PN, Erickson BJ, D'Angelo J, Ma K, Romeo AA (2019). Epidemiology of shoulder surgery among professional baseball players. Am J Sports Med.

[3] Chambers CC, Lynch TS, Gibbs DB (2017). Superior Labrum Anterior-Posterior Tears in the National Football League. Am J Sports Med.

[4] Rossy W, Sanchez G, Sanchez A, Provencher MT (2016). Superior labral anterior-posterior (SLAP) tears in the military. Sports Health.

[5] Brockmeyer M, Tompkins M, Kohn DM, Lorbach O (2016). SLAP lesions: a treatment algorithm. Knee Surg Sports Traumatol Arthrosc.

[6] Sullivan S, Hutchinson ID, Curry EJ, Marinko L, Li X (2019). Surgical management of type II superior labrum anterior posterior (SLAP) lesions: a review of outcomes and prognostic indicators. Phys Sportsmed.

[7] Erickson BJ, Jain A, Abrams GD, Nicholson GP, Cole BJ, Romeo AA, Verma NN (2016). Slap lesions: trends in treatment. Arthroscopy.

[8] Gorantla K, Gill C, Wright RW (2010). The outcome of type II SLAP repair: a systematic review. Arthroscopy.

[9] Alpert JM, Wuerz TH, O'Donnell TF, Carroll KM, Brucker NN, Gill TJ (2010). The effect of age on the outcomes of arthroscopic repair of type II superior labral anterior and posterior lesions. Am J Sports Med.

[10] Ek ET, Shi LL, Tompson JD, Freehill MT, Warner JJ (2014). Surgical treatment of isolated type II superior labrum anterior-posterior (SLAP) lesions: repair versus biceps tenodesis. J Shoulder Elbow Surg.

[11] de Sa D, Arakgi ME, Lian J, Crum RJ, Lin A, Lesniak BP (2019). Labral repair versus biceps tenodesis for primary surgical management of type II superior labrum anterior to posterior tears: a systematic review. Arthroscopy.

[12] Griffin JW, Cvetanovich GL, Kim J (2019). Biceps tenodesis is a viable option for management of proximal biceps injuries in patients less than 25 years of age. Arthroscopy.

[13] Provencher MT, McCormick F, Peebles LA (2019). Outcomes of primary biceps subpectoral tenodesis in an active population: a prospective evaluation of 101 patients. Arthroscopy.

[14] Schrøder CP, Skare Ø, Reikerås O, Mowinckel P, Brox JI (2017). Sham surgery versus labral repair or biceps tenodesis for type II SLAP lesions of the shoulder: a three-armed randomised clinical trial. Br J Sports Med.

[15] Boileau P, Parratte S, Chuinard C, Roussanne Y, Shia D, Bicknell R (2009). Arthroscopic treatment of isolated type II SLAP lesions: biceps tenodesis as an alternative to reinsertion. Am J Sports Med.

[16] Provencher M, McCormick F, LeClere LE, Solomon DJ, Dewing CB (2014). Outcomes of primary biceps sub-pectoral tenodesis in an active population: a prospective evaluation of 101 patients. Orthop J Sports Med.

[17] Johannsen AM, Macalena JA, Carson EW, Tompkins M (2013). Anatomic and radiographic comparison of arthroscopic suprapectoral and open subpectoral biceps tenodesis sites. Am J Sports Med.

[18] Frantz TL, Shacklett AG, Martin AS, Barlow JD, Jones GL, Neviaser AS, Cvetanovich GL (2021). Biceps tenodesis for superior labrum anterior-posterior tear in the overhead athlete: a systematic review. Am J Sports Med.

[19] Jacxsens M, Granger EK, Tashjian RZ (2018). Clinical and sonographic evaluation of subpectoral biceps tenodesis with a dual suture anchor technique demonstrates improved outcomes and a low failure rate at a minimum 2-year follow-up. Arch Orthop Trauma Surg.

[20] Friedman JL, FitzPatrick JL, Rylander LS, Bennett C, Vidal AF, McCarty EC (2015). Biceps tenotomy versus tenodesis in active patients younger than 55 years: is there a difference in strength and outcomes?. Orthop J Sports Med.

